# Lending a helping hand: the complementary role of actigraphy in the assessment of central disorders of hypersomnolence

**DOI:** 10.1093/sleep/zsae228

**Published:** 2024-09-27

**Authors:** Jesse D Cook, Cathy Goldstein, David T Plante

**Affiliations:** Department of Psychiatry, University of Wisconsin-Madison, Madison, WI, USA; Department of Neurology, Sleep Disorders Center, University of Michigan-Ann Arbor, Ann Arbor, MI, USA; Department of Psychiatry, University of Wisconsin-Madison, Madison, WI, USA; Department of Psychology, University of Wisconsin-Madison, Madison, WI, USA

Narcolepsy and idiopathic hypersomnia (IH) are closely related central disorders of hypersomnolence (CDH) that share the cardinal symptom of excessive daytime sleepiness (EDS) [1]. The objective measurement of nocturnal and diurnal sleep plays a crucial role in the assessment of these disorders. Diagnostic criteria center around objective testing with in-laboratory, nocturnal polysomnography (PSG) followed by a multiple sleep latency test (MSLT) the following day. However, actigraphy also plays an important role in the diagnostic process. Actigraphy estimates sleep and wake periods based on motor activity derived from accelerometry, and is widely applied across clinical sleep medicine [2, 3]. In the context of CDH assessment, actigraphy has multiple purposes. It can rule out insufficient sleep duration and circadian rhythm sleep–wake disorders as an underlying cause of EDS, while also ensuring adequate sleep duration preceding PSG and MSLT [2]. In addition, results from actigraphy can also be utilized in the diagnosis of IH based on 24-h total sleep time (TST; ≥660 min) in lieu of positive results from the PSG/MSLT or 24-h polysomnographic recordings [1]. Despite the importance of actigraphy in these domains, few studies have evaluated the accuracy of TST estimations from actigraphy in patients with CDH [4–8], with none of the existing literature assessing the ability of actigraphy to measure daytime sleep in CDH. Addressing this knowledge gap is paramount for appropriately guiding the use of actigraphy as a tool in the diagnostic process for CDH.

The investigation by Biscarini et al. is a critical evaluation of actigraphy’s ability to measure daytime sleep in CDH [9]. A cross-sectional sample of patients diagnosed with narcolepsy (*n* = 28) and IH (*n* = 16) completed a multiple day-and-night, in-laboratory protocol that included 2 days of continuous PSG, followed by a five-nap MSLT on the third day [9]. Some participants (*n* = 13) also completed a maintenance of wakefulness test (MWT) on the fourth day. All participants concurrently wore the Micro Motionlogger Watch actigraph (Ambulatory Monitoring, Inc., Ardsley, NY, USA) across the protocol. Actigraphy data were scored in 30-s epochs based on three widely utilized legacy scoring algorithms: Cole–Kripke (CK) [10], Sadeh [11], and the University of California San Diego (UCSD) [12]. Analyses focused on concordance between PSG and actigraphy for daytime, nighttime, and 24-h sleep, as well as actigraphy’s ability to identify sleep occurrence and estimate mean sleep latency (MSL) during both MSLT and MWT.

The primary results evidenced a significant overestimation of daytime TST during continuous PSG by actigraphy in both disorders (i.e., narcolepsy and IH), regardless of the scoring algorithm. Although actigraphy’s general propensity to overestimate TST is well established [13], the authors also suggested that increased daytime rest as a consequence of EDS may independently contribute to these findings. Notably, the disagreement between PSG and actigraphy for daytime TST was greater in IH, relative to narcolepsy, which may reflect a greater propensity for rest or stationary behavior during wakefulness. The authors posit that this could be a marker of phenotypic differences between the disorders (e.g., sleep inertia), cautiously proposing that the degree of bias between PSG and actigraphy for daytime TST could potentially be a distinguishing characteristic of IH from narcolepsy. Importantly, the narcolepsy sample was predominantly type 1 (NT1; 78.6%). Thus, the degree to which IH and narcolepsy type 2 (NT2) differ in the disagreement between PSG and actigraphy for daytime TST is unclear. Future research aimed at clarifying this dynamic is warranted, as these parameters may aid clinicians in differentiating non-cataplectic CDH (i.e., IH vs. NT2) which is diagnostically complex and challenging [14].

In terms of nighttime TST, actigraphy TST estimations were statistically comparable to PSG in IH, while significant underestimations of nighttime TST were observed in narcolepsy for CK (−27.8 min) and Sadeh (−73.7 min) algorithms, but not UCSD (−5.04 min). These results highlight the potential of actigraphy to capture increased nighttime motor activity that is a signature of narcolepsy, particularly NT1. Also, the vulnerability to nighttime TST underestimations in narcolepsy may be clinically relevant. This could potentially bias the evaluation of a patient’s habitual nighttime TST preceding PSG/MSLT, leading to a spurious conclusion that habitual sleep duration is inadequate. Furthermore, these results exemplify the estimation variability between scoring algorithms used in actigraphy [15]. Reported estimates of 24-h TST provide another example of sizeable, clinically relevant variations in accuracy between the three widely utilized actigraphy scoring algorithms. Using the entire sample, UCSD significantly overestimated (86.9 min), while nonsignificant overestimations and underestimations were produced by CK (24.7 min) and Sadeh (−10.4 min), respectively. In IH, all algorithms overestimated 24-h TST, but the magnitude of overestimation varied greatly (34.8–119 min). Since 24-h TST is a diagnostic criterion for IH [1], the authors appropriately note the need for caution when applying actigraphy to assess 24-h TST in IH. The sizeable differences in overestimations between algorithms also underscore the need for actigraphy scoring standardization to enhance diagnostic consistency between clinical sites. Moreover, research aimed at developing a protocol to personalize actigraphy scoring could be potentially fruitful for increasing the accuracy of actigraphy-derived TST estimations in CDH patients.

Considering the importance of correctly measuring sleep latency in CDH, establishing convenient, actigraphy-based approaches to assess sleep latency could greatly benefit patient screening and monitoring. Mirroring other analyses, results were mixed and varied depending on the algorithm used as well asthe disorder; however, sleep latency estimates on nocturnal PSG and MSL on the MSLT were comparable when applying the CK algorithm in IH and the UCSD algorithm in narcolepsy, respectively. Interestingly, better MSL accuracy was observed during the MWT, relative to the MSLT, which the authors note may reflect inherent differences in the two tests (i.e., try to fall asleep versus stay awake) and the magnitude of motor activity preceding sleep onset. However, these preliminary findings must be interpreted with caution, especially considering the small sample size in the MWT analyses. Nevertheless, there is a need for convenient approaches to assess sleep propensity that can be administered habitually and repeatedly. Actigraphy may be able to serve this role; yet, strict reliance on accelerometry may ultimately prove insufficient. Future research that explores the capabilities of multisensor wearables, particularly those including electroencephalography, as tools to measure sleep latency is warranted. Advances in this area could evolve into home-based protocols that complement the gold standard, in-laboratory evaluation, potentially improving clinical care by enhancing access to assessments, serving as a screening tool, and enabling convenient monitoring during treatment.

In summary, Biscarini et al. performed a foundational investigation of actigraphy’s concordance with PSG for measuring daytime sleep in CDH, highlighting a tendency for overestimations of daytime TST in both narcolepsy and IH. The results also draw attention to the potential for clinically relevant underestimations of nighttime TST in narcolepsy and overestimations of 24-h TST in IH, while also highlighting the need for scoring standardization and/or personalization to enhance diagnostic precision and accuracy from actigraphy. In addition, actigraphy showed some potential as a measure of sleep latency, but sole reliance on accelerometry is likely to be insufficient for accurate and reliable sleep latency measurement. Future work evaluating the capabilities of multisensor wearables to estimate daytime, nighttime, and nap-specific sleep and sleep latency in CDH is warranted. In the interim, the manuscript by Biscarini et al. is a key step forward as the field works to clarify the role of actigraphy in CDH assessment.
